# The Scaffolding Protein SYD-2/Liprin-α Regulates the Mobility and Polarized Distribution of Dense-Core Vesicles in *C. elegans* Motor Neurons

**DOI:** 10.1371/journal.pone.0054763

**Published:** 2013-01-24

**Authors:** Patricia R. Goodwin, Peter Juo

**Affiliations:** 1 Department of Molecular Physiology and Pharmacology, Sackler School of Graduate Biomedical Sciences, Tufts University School of Medicine, Boston, Massachusetts, United States of America; 2 Graduate Program in Neuroscience, Sackler School of Graduate Biomedical Sciences, Tufts University School of Medicine, Boston, Massachusetts, United States of America; Columbia University, United States of America

## Abstract

The polarized trafficking of axonal and dendritic components is essential for the development and maintenance of neuronal structure and function. Neuropeptide-containing dense-core (DCVs) vesicles are trafficked in a polarized manner from the cell body to their sites of release; however, the molecules involved in this process are not well defined. Here we show that the scaffolding protein SYD-2/Liprin-α is required for the normal polarized localization of Venus-tagged neuropeptides to axons of cholinergic motor neurons in *C. elegans*. In *syd-2* loss of function mutants, the normal polarized localization of INS-22 neuropeptide-containing DCVs in motor neurons is disrupted, and DCVs accumulate in the cell body and dendrites. Time-lapse microscopy and kymograph analysis of mobile DCVs revealed that *syd-2* mutants exhibit decreased numbers of DCVs moving in both anterograde and retrograde directions, and a corresponding increase in stationary DCVs in both axon commissures and dendrites. In addition, DCV run lengths and velocities were decreased in both axon commissures and dendrites of *syd-2* mutants. This study shows that SYD-2 promotes bi-directional mobility of DCVs and identifies SYD-2 as a novel regulator of DCV trafficking and polarized distribution.

## Introduction

There are many similarities between DCVs and synaptic vesicles (SVs). For example, both DCVs and SVs can be localized in a polarized manner at presynaptic sites and are transported to axons by the kinesin UNC-104/KIF1A in *C. elegans*, *Drosophila* and rodent cultured neurons [1]–[6]. Recent studies in *C. elegans* motor neurons showed that the polarized trafficking of both SVs and DCVs are regulated by cyclin-dependent kinase CDK-5 which functions to prevent inappropriate trafficking of these vesicles to dendrites [7], [8]. Despite these similarities, SVs and DCVs also have distinct properties. For example, SVs are filled with chemical neurotransmitters and are typically clustered and released at active zones [9], [10]. In contrast, DCVs contain neuropeptides, neurotrophins and peptide hormones, which are involved in modulating synaptic transmission and plasticity [11]–[13]. DCVs are excluded from active zones [14], and exocytosis of DCVs typically requires higher frequency stimulation for release than SVs [15], [16]. After release, SVs can be refilled with neurotransmitter at synaptic terminals and undergo multiple rounds of exocytosis and endocytosis. In contrast, DCVs are not recycled in this manner, and must be replenished by newly generated DCVs from the cell body. DCVs are packaged with their protein cargo at the *trans*-golgi network and undergo a complex biogenesis process, prior to being transported from the cell body to their sites of release [17]–[21]. While several studies have focused on understanding how SVs are trafficked and localized to presynaptic sites [8], [22]–[30], the molecules that mediate the targeting of DCVs to their release sites in axons are not well understood. Given that DCVs have different trafficking requirements and are more broadly distributed at release sites than SVs, it is not clear if previously defined regulators of SV trafficking and clustering are also required to localize DCVs to their release sites.

Here we investigated whether SYD-2/Liprin-α regulates the polarized trafficking of DCVs to axons. SYD-2/Liprin-α is a multifunctional scaffolding protein that localizes to the active zone and is required for presynaptic terminal differentiation in *C. elegans* and *Drosophila* [31]–[35]. SYD-2/Liprin-α can oligomerize and interact with other presynaptic components to regulate active zone assembly and morphology in *C. elegans* and *Drosophila* [31], [32], [36]–[41]. For example, in HSN neurons, active zone components and SVs fail to localize at presynaptic sites and instead mis-localize to non-synaptic sites in the axon of *syd-2* mutants [36], [37].

SYD-2/Liprin-α has also been implicated in regulating intracellular transport. SYD-2/Liprin-α has been shown to interact with kinesin motors and regulate the movement characteristics of those motors and their associated cargo [42]–[45]. For example, in *Drosophila*, Liprin-α associates with kinesin-1 and promotes the anterograde flux and run length of its SV cargo [43]. In *C. elegans*, SYD-2 binds and clusters kinesin UNC-104/KIF1A, and promotes its anterograde flux and velocity [45]. Mammalian SYD-2/Liprin-α has been shown to bind UNC-104/KIF1A *in vitro* but the function of this interaction in mammalian neurons has not been studied [44]. Finally, Liprin-α can associate with the PDZ scaffold GRIP1 and promote the trafficking of postsynaptic proteins such as glutamate receptors in cultured rodent hippocampal neurons [46]–[49]. These studies show that in addition to promoting synapse formation, SYD-2 can interact with motors and regulate the trafficking of motors and multiple types of cargo.

A recent synaptic profiling study implicated SYD-2 as a regulator of DCV localization. This systematic study compared the protein composition of motor neuron synapses across a panel of genetic mutant backgrounds and showed that *syd-2* mutants had decreased amounts of the neuropeptide INS-22 at presynaptic sites [50], however, the reason for this decrease is not known. Here, we investigate the mechanism by which SYD-2 affects the abundance of neuropeptides in motor neuron axons. We confirm that SYD-2 regulates the abundance of INS-22 at presynaptic sites and show that SYD-2 also affects the levels of another neuropeptide NLP-21 in axons. We use quantitative fluorescence analysis to examine the distribution of these Venus-tagged neuropeptides in motor neuron axons and dendrites and show that SYD-2 is required for the normal polarized distribution of DCVs to axons. Furthermore, time-lapse microscopy of mobile DCVs in motor neurons reveals that SYD-2 promotes the overall mobility of DCVs in axon commissures and dendrites. This study identifies SYD-2 as a novel regulator of DCV trafficking and polarized distribution.

## Materials and Methods

### Strains

Strains were maintained on OP50 *E. coli* at 20°C as described by Brenner et al. (1974) [51]. The following strains were used in this study: N2 Bristol, *nuIs195 (Punc-129::ins-22::venus), nuIs183(Punc129::nlp-12::Venus); nuIs168(Punc-129::rab-3::Venus), yuEx46(Punc-129::unc-9::gfp)* (gift from Lars Dreier, University of California, Los Angeles), *pzEx141(Punc-129::syd-2), syd-2(ju37), syd-2(ok217), cdk-5(gm336), dhc-1(js319).* The *ju37* mutation in *syd-2* is a point mutation which results in a premature stop codon in the coiled-coil domain (glutamine 397)(Zhen 1999) and a truncated protein [45]. The *ok217* mutation in *syd-2* is a deletion of the N-terminal coiled-coil domains, which results in an ochre stop codon and no detectable protein product [45].

### Constructs, Transgenes, and Germline Transformation

Plasmids were generated using standard cloning techniques and details are available upon request. Briefly, genomic *syd-2* (gift from Mei Zhen, University of Toronto) was tagged at the N-terminus with mRFP using engineered Not I sites and subcloned under the control of the *Punc-129* promoter to create *Punc-129::mRFP::syd-2* (KP#1132). The mRFP was subcloned from KP#1132 (gift from Dave Simon and Josh Kaplan) to create *Punc-129::syd-2* (FJ#82). FJ#82 was injected at 25 ng/µl together with a coinjection marker (*Pmyo-2::NLS-mCherry*) to create *pzEx141.*

### Fluorescent Microscopy and Quantification

All imaging was performed using a Zeiss M1 Axioimager microscope. For all static imaging experiments, young adult animals were immobilized with 30 mg/mL 2,3-Butanedione monoxamine (Sigma Aldrich) for 5–7 minutes and mounted on 2% agarose pads prior to imaging. Images were captured using a Zeiss 100X PlanApo objective (NA 1.4) and an Orca-ER (Hamamatsu) CCD camera. Maximum intensity projections were generated from Z-series stacks and line scans of fluorescent puncta were acquired using Metamorph (v7.1) software (Molecular Devices). For quantitative analyses of fluorescent ventral and dorsal nerve cord puncta, maximum intensity projections of Z series stacks (1 µm total depth) were generated. Exposure settings and gain were selected to fill the 12-bit dynamic range to avoid saturation and were kept constant for all images of a given fluorescent marker. Because the fluorescent neuropeptide markers were much dimmer in the VNC, we had to use different acquisition settings to image the VNC and DNC. Dorsal nerve cord (axon) images were taken using 1×1 binning and 50× gain, while ventral nerve cord (dendrite) images were taken using 2×2 binning and 100× gain. Consequently, the axonal and dendritic images cannot be directly compared. FluoSphere fluorescent beads (Invitrogen) were imaged every day to correct for variation in microscope light bulb intensity. All images were acquired from animals with their VNC or DNC oriented towards the objective. Laterally-oriented animals were excluded from analysis.

The *unc-129* promoter drives expression of genes in DA motorneurons, which have processes projecting towards the head, and DB neurons, whose processes project towards the tail. DA6 is the most posterior DA neuron with visible expression of fluorescent markers driven by the *unc-129* promoter [7]. Thus, all fluorescent signal posterior to the DA6 neuron cell body is derived only from DB neurons. To isolate signal originating from only DB axons in the DNC, we drew line scans along the DNC posterior to the site of entry of the DA6 commissure. To isolate signal originating from only DB dendrites in the VNC, we drew line scans posterior to the DA6 cell body, as previously described [7].

Line scans of VNC and DNC puncta were obtained using MetaMorph (v6.0) and were subsequently analyzed in Igor Pro (v5) using custom written software, as previously described [52]. Puncta intensity and density were calculated from each image. Puncta intensity is calculated as the fractional increase in peak fluorescence of each puncta over fluorescent bead intensity for that imaging day. Puncta density is calculated as the average number of puncta per 10 µm of VNC or DNC. Puncta density measurements of DCVs in the VNC dendrites likely overestimate the density of DCVs, especially in wild type animals where dendritic DCVs are very sparse, or in some cases, absent. Our analysis software requires at least two puncta to demarcate the region to be analyzed and ensure the cord is in focus. Thus, VNC images with fewer than two in focus DCVs were discarded from analysis.

Images of INS-22::Venus in DB6 cell bodies were acquired from animals with their lateral or ventral sides oriented towards the objective, and Z stacks were taken to a depth of 2 µm. The average fluorescence of three fluorescent patches per cell body was measured using Metamorph software, then corrected for daily bead values and analyzed in Microsoft Excel.

To quantify the fluorescence of INS-22::Venus in coelomocytes, we imaged a posterior coelomocyte near the dorsal nerve cord between the DA6 and DB7 cell bodies. Z-stacks were taken to a depth of 2 µm and the average fluorescence of four fluorescent patches per cell body was measured using Metamorph software, then corrected for daily bead values and analyzed in Microsoft Excel, as previously described [53]. Changes in puncta intensity and density were analyzed for statistical significance using Student’s t test for two genotype comparisons and Tukey-Kramer for multiple genotype comparisons.

### Time-lapse Microscopy

Young adult worms were paralyzed in 360 µg/mL Levamisol (Sigma) dissolved in M9 buffer for 7 to 9 minutes prior to time-lapse. Animals were mounted on 2% agarose pads containing 360 µg/ml Levamisol. Time-lapse images of INS-22::Venus in dendrites were acquired from a region of the VNC between the DA6 and DB7 cell bodies. Because DB processes project towards the posterior, all movements towards the tail were designated as anterograde and all movements towards the head were designated as retrograde. Time-lapse imaging of DB6 axon commissures were acquired from animals with their ventral or lateral side oriented towards the objective. Line scans were drawn from the DB6 cell body to the last in-focus INS-22::Venus punctum in the commissure.

Time-lapse images were acquired at 4 Hz for 20 seconds and used to generate kymographs in MetaMorph (v7.1). Mobile INS-22::Venus puncta on these kymographs were traced in order to calculate direction of puncta movement and velocity. Each mobile punctum was traced during its longest uninterrupted period of movement. Puncta were defined as mobile if they moved distances greater than twice their own width and at velocities greater than 0.1 µm/s. Puncta that changed direction were traced in both directions. Kymograph traces were analyzed using Microsoft Excel and histograms of puncta velocity were generated using IgorPro (v5). Average anterograde and retrograde velocity and the number of stationary, anterogradely, and retrogradely moving puncta were calculated for each kymograph (i.e., per worm) and these data were compiled for each genotype and analyzed in Microsoft Excel.

## Results

### SYD-2 Regulates the Abundance of DCVs in Motor Neuron Axons

We investigated the role of *syd-2* in DCV trafficking in DA and DB subclasses of cholinergic motor neurons. DA and DB motor neurons have a simple polarized morphology with a cell body and dendrite in the ventral nerve cord (VNC) and an axon that extends across the body (axon commissure) and along the dorsal nerve cord (DNC) where it makes *en-passant* synapses onto muscle cells [54]. DA neuron axons and dendrites extend from the cell body towards the anterior, while DB neuron processes extend towards the posterior of the animal. We used the *unc-129* promoter to express fluorescently tagged proteins in a subset of 10 DA and DB neurons as previously described [7](see Materials and Methods). In order to visualize DCVs in these neurons, we expressed Venus-tagged Insulin-like-protein-22 (INS-22::Venus) or Neuropeptide-like-protein-21 (NLP-21::Venus) under control of the *unc-129* promoter [4], [53]. Both INS-22 and NLP-21 regulate synaptic transmission at the neuromuscular junction [4]. Fluorescently-tagged versions of these neuropeptides have been widely used to study DCV trafficking in *C. elegans* [4], [7], [18], [20], [21], [50], [53]. In DA and DB motor neurons, INS-22::Venus and NLP-21::Venus have a polarized distribution where they are localized to presynaptic sites in the axon and are largely excluded from the dendrite [4], [7]. Trafficking of both NLP-21 and INS-22 require the kinesin UNC-104/KIF1A, as has been shown for endogenous neuropeptides and other DCV markers [1], [2], [4]–[6].

**Figure 1:**
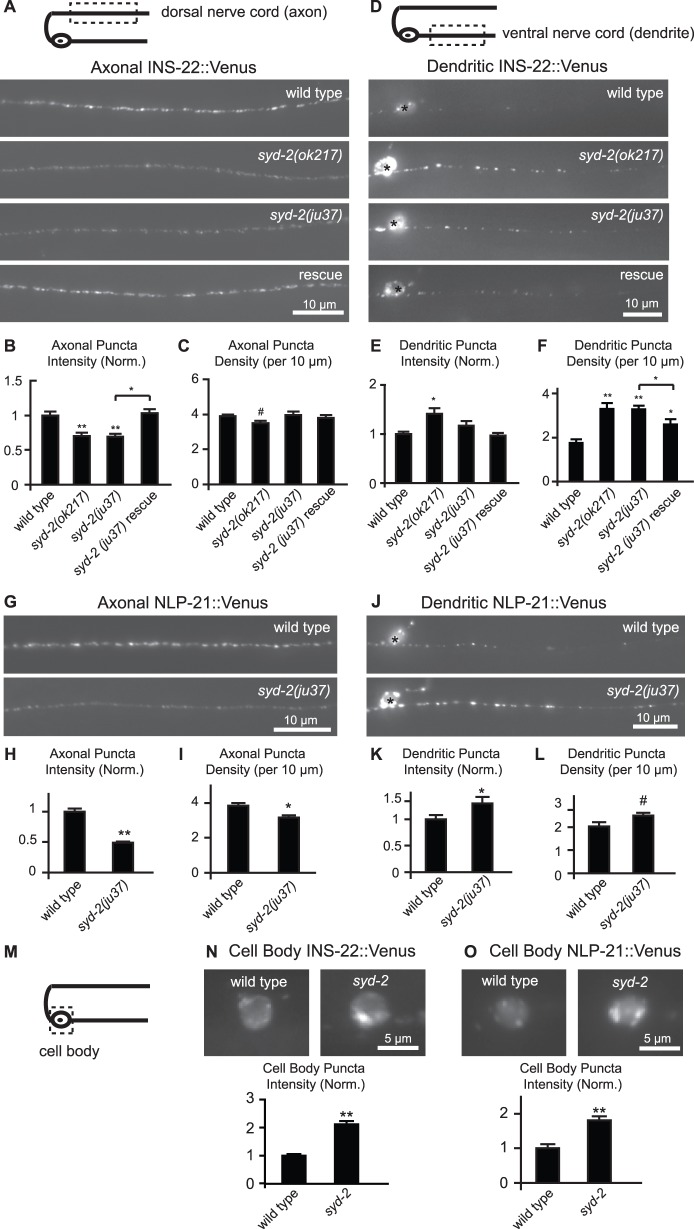
*syd-2* regulates the polarized distribution of neuropeptides in DB motorneurons. (A) Schematic diagram of a DB motor neuron with its axon in the dorsal nerve cord (DNC) and its dendrite in the ventral nerve cord (VNC)(top panel). This diagram is oriented with the anterior of the animal to the left for this and all subsequent figures. The boxed region denotes that the axon was imaged for data presented in panels (A–C, G–I). Representative images of INS-22::Venus in the axon of wild type, *syd-2(ok217)*, *syd-2(ju37)*, and *Punc-129::syd-2;syd-2(ju37)* rescue animals (bottom panels). (B–C) Quantification of INS-22::Venus puncta intensity (B) and density (C) in the axons of wild-type (n = 33), *syd-2(ok217)*(n = 24), *syd-2(ju37)(*n = 17), and *syd-2* rescue animals (n = 14). (D) Schematic diagram of DB motor neuron (top panel). The boxed region denotes that the dendrite was imaged for panels (D–F, J–L). Representative images of INS-22::Venus in the DB dendrite of wild-type, *syd-2(ok217), syd-2(ju37),* and *Punc-129::syd-2;syd-2(ju37)* rescue animals (bottom panel). (E–F) Quantification of INS-22::Venus puncta intensity (E) and density (F) in the dendrites of wild-type (n = 44), *syd-2(ok217)*(n = 31), *syd-2(ju37)*(n = 30), and *Punc-129::syd-2;syd-2(ju37)* rescue animals(n = 19). (G) Representative images of NLP-21::Venus in the DB axons of wild type and *syd-2(ju37)* mutant animals. (H-I) Quantification of NLP-21::Venus puncta intensity (H) and density (I) in the axons of wild type (n = 12) and *syd-2(ju37)*(n = 18) mutant animals. (J) Representative images of NLP-21::Venus in dendrites of wild type and *syd-2(ju37)* mutant animals. (K–L) Quantification of NLP-21::Venus puncta intensity (K) and density (L) in the dendrites of wild-type (n = 20) and *syd-2(ju37)*(n = 22) animals. For this and all subsequent figures, error bars denote standard error from the mean (SEM) and cell bodies in the images are marked by an asterisk. Values that differ significantly (Tukey-Kramer (B, C, E and F); Student’s *t* test (H, I,K, and L) from wild type (marked by asterisks above each bar) or from other genotypes (comparisons marked by brackets) are denoted on the graphs (#p≤0.05, *p≤0.01, **p≤0.001). (M) Schematic diagram of a DB neuron. The boxed region denotes that the cell body was imaged for data presented in panels (N-O). (N) Representative images of INS-22::Venus in the motor neuron cell bodies of wild-type and *syd-2(ok217)* mutant animals. The graph shows quantification of somatic INS-22::Venus puncta fluorescence intensity for wild-type (n = 24) and *syd-2(ok217)*(n = 22) mutant animals. (O) Representative images of NLP-21::Venus in the motor neuron cell bodies of wild type and *syd-2(ju37)* mutant animals. The graph shows quantification of somatic NLP-21::Venus puncta fluorescence intensity for wild type (n = 7) and *syd-2(ju37)*(n = 10) mutant animals. Values that differ significantly from wild type (Student’s *t* test) are denoted on the graphs (**p≤0.001).

To determine if *syd-2* regulates the polarized distribution of DCVs, we used quantitative fluorescence microscopy to examine the localization of INS-22::Venus and NLP-21::Venus in DB neurons in two *syd-2* loss-of-function mutants, *ju37* and *ok217*. Both *syd-2(ju37)* and *syd-2(ok217)* alleles contain mutations that result in premature stop codons [35], [45](see Materials and Methods). We quantified the puncta intensity and density of fluorescently-tagged markers using custom written software [7], [52](see Materials and Methods). Because INS-22::Venus puncta can represent clusters of multiple DCVs [7], a change in the number of DCVs at a presynaptic site can result in changes in either puncta intensity or density. We found that *syd-2(ju37)* and *syd-2(ok217)* loss-of-function mutants had a significant reduction (30%) in INS-22::Venus puncta fluorescence intensity in DB axons compared to wild type controls (Fig. 1A, B), consistent with previous studies using the *ju37* allele [50]. Similarly, we found that the puncta fluorescence intensity of NLP-21::Venus was reduced by about 50% in *syd-2(ju37)* mutant axons (Fig. 1G, H). Analysis of INS-22::Venus and NLP-21::Venus in *syd-2* mutants also revealed that both neuropeptides had a small but significant decrease in puncta density in DB axons (Fig. 1C, I). However, the decrease in INS-22 density in axons did not reach statistical significance in all experiments (i.e., in the *syd-2;cdk-5* double mutant analysis described below).

To determine if SYD-2 acts cell autonomously in DA/DB motor neurons to regulate neuropeptide abundance in axons, we performed cell-type specific rescue experiments. We expressed wild-type *syd-2* cDNA under the control of the DA/DB motor neuron-specific promoter P*unc-129* to restore SYD-2 function only in these motor neurons. Expression of *syd-2* in DA/DB neurons was sufficient to rescue the decrease in INS-22::Venus fluorescence in the axons of *syd-2(ju37)* mutants (Fig. 1A, B), indicating that SYD-2 functions in these cholinergic motor neurons to regulate neuropeptide abundance in axons.

**Figure 2:**
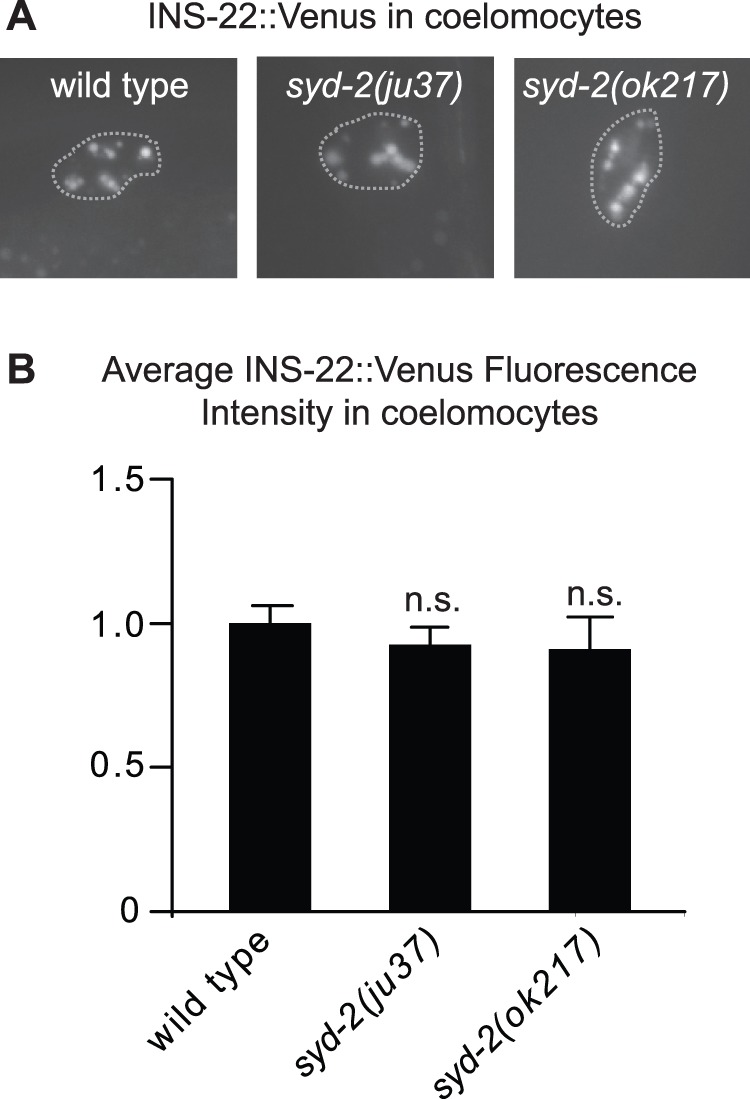
Analysis of INS-22::Venus accumulation in coelomocytes of *syd-2* mutants. (A) Representative images of INS-22::Venus accumulation in coelomocytes (outlined by a dotted line) of wild type, *syd-2(ju37)* and *syd-2(ok217)* mutant animals. (B) Quantification of INS-22::Venus fluorescence intensity in coelomocytes of wild type (n = 39), *syd-2(ju37)*(n = 27) and *syd-2(ok217)*(n = 14) mutant animals. Average intensity (normalized)±standard error are shown. Values that did not differ significantly from wild type (Student’s *t* test) are denoted on the graph by n.s. (p>0.05).

Decreased INS-22::Venus fluorescence in the axon could result from either reduced trafficking of neuropeptides to presynaptic sites or enhanced release of neuropeptides from axons into the body cavity. Neuropeptide release can be indirectly measured in *C. elegans* by measuring the amount of fluorescently-tagged neuropeptides that accumulate in scavenger cells called coelomocytes, which endocytose secreted proteins from the body cavity [53]. We found no difference in the amount of INS-22::Venus in *syd-2(ju37)* and *syd-2(ok217)* mutant coelomocytes compared to wild type controls (Fig. 2), suggesting that increased release of neuropeptides is unlikely to account for the decrease in INS-22::Venus in *syd-2* mutant axons.

### SYD-2 Regulates the Polarized Distribution of DCVs in DB Motor Neurons

SYD-2 promotes the anterograde trafficking of synaptic vesicles in axons in *Drosophila* and *C. elegans* [43], [45]. To determine if DCV trafficking is also regulated by SYD-2, we examined DB motor neuron cell bodies and dendrites for accumulation of Venus-tagged neuropeptides. We found that the amount of INS-22::Venus and NLP-21::Venus fluorescence increased significantly in DB6 cell bodies of *syd-2* mutant animals relative to wild type controls (Fig. 1M–O), suggesting that *syd-2* is required for trafficking of DCVs out of the cell body. We also found an increased abundance of DCVs in *syd-2* mutant DB dendrites (Fig. 1D–F, J–L). Quantitative analysis of INS-22::Venus fluorescence in the VNC revealed an increase in INS-22::Venus density in both *syd-2(ju37)* and *syd-2(ok217)* mutant dendrites (Fig. 1D, F). *syd-2(ok217)* null mutants also had brighter INS-22::Venus puncta in the dendrite relative to wild type controls (Fig. 1D, E). We saw no significant change in INS-22::Venus puncta intensity in *syd-2(ju37)* mutant dendrites (Fig. 1E). The weaker effect of the *ju37* allele might suggest that *ok217* is a stronger *syd-2* loss of function allele than *ju37* (see Materials and Methods). We also found that NLP-21::Venus fluorescence intensity and density were increased in the dendrites of *syd-2(ju37)* mutant animals relative to wild type controls (Fig. 1J–L). The increase in INS-22::Venus density observed in *syd-2(ju37)* mutant dendrites was partially rescued by expression of wild-type *syd-2* cDNA under the control of the *unc-129* promoter (Fig. 1F). Together, these data indicate that SYD-2 functions in motor neurons to regulate the polarized distribution of DCVs to axons.

### SYD-2 and CDK-5 Function in Parallel Pathways to Regulate Polarized DCV Distribution

**Figure 3:**
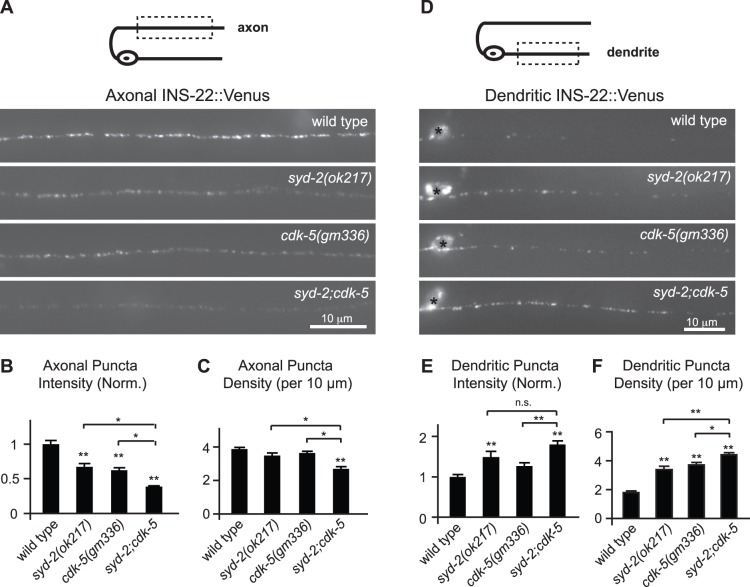
*syd-2* and *cdk-5* regulate the polarized distribution of neuropeptides through separate genetic pathways. (A) Schematic diagram of a DB motor neuron (top panel). The boxed region denotes that the axon was imaged for panels (A–C). Representative images of INS-22::Venus in the axons of wild type, *syd-2(ok217*), *cdk-5(gm336),* and *syd-2(ok217);cdk-5(gm336)* double mutant animals (bottom panel). (B–C) Quantification of INS-22::Venus puncta intensity (B) and density (C) in the axons of wild type (n = 32), *syd-2(ok217)*(n = 24), *cdk-5(gm336)*(n = 35), and *syd-2;cdk-5* double mutant (n = 19) animals. (D) Schematic diagram of a DB motor neuron (top panel). The boxed region denotes that the dendrite was imaged for panels D-F. Representative images of INS-22::Venus in the dendrites of wild type, *syd-2(ok217*), *cdk-5(gm336),* and *syd-2(ok217);cdk-5(gm336)* double mutant animals (bottom panel). (E–F) Quantification of INS-22::Venus puncta intensity (E) and density (F) in the dendrites of wild type(n = 44), *syd-2(ok217)*(n = 26), *cdk-5(gm336)*(n = 31), and *syd-2;cdk-5* double mutant (n = 32) animals. Values that differ significantly (Tukey-Kramer) from wild type (marked by asterisks above each bar) or from other genotypes (comparisons marked by brackets) are denoted on the graphs (#p≤0.05, *p≤0.01, **p≤0.001). Values that did not differ significantly (p>0.05) are denoted by n.s.

We previously showed that cyclin-dependent kinase 5 (CDK-5) is required for polarized trafficking of DCVs to axons in DA/DB motor neurons [7]. Similar to *syd-2* mutants, *cdk-5* mutants exhibit a 40% reduction in INS-22::Venus puncta intensity in DB neuron axons and about an 80% increase in INS-22::Venus puncta density in DB neuron dendrites (Fig. 3) [7]. Since both *cdk-5* and *syd-2* mutants have similar defects in the polarized distribution of DCVs, we tested whether these two genes function in the same pathway by examining the distribution of INS-22::Venus in *syd-2;cdk-5* double mutants. If these two genes act in the same pathway, then the change in the polarized distribution of DCVs in *syd-2;cdk-5* double mutants should be identical to either single mutant. However, in axons we found that *syd-2;cdk-5* double mutants had a dramatic decrease in INS-22::Venus puncta fluorescence compared to wild type controls and that this decrease was significantly different from either *syd-2* or *cdk-5* single mutants (Fig. 3A–C). Similarly, in dendrites we found that *syd-2;cdk-5* double mutants had an additive increase in INS-22::Venus puncta density compared to wild type controls and that this increase was significantly different from either *syd-2* or *cdk-5* single mutants (Fig. 3D, F). These additive effects suggest that SYD-2 functions in a novel, CDK-5-independent pathway to regulate the polarized distribution of DCVs.

### Axonal and Dendritic Markers are Relatively Normal in *syd*-2 Mutants

**Figure 4:**
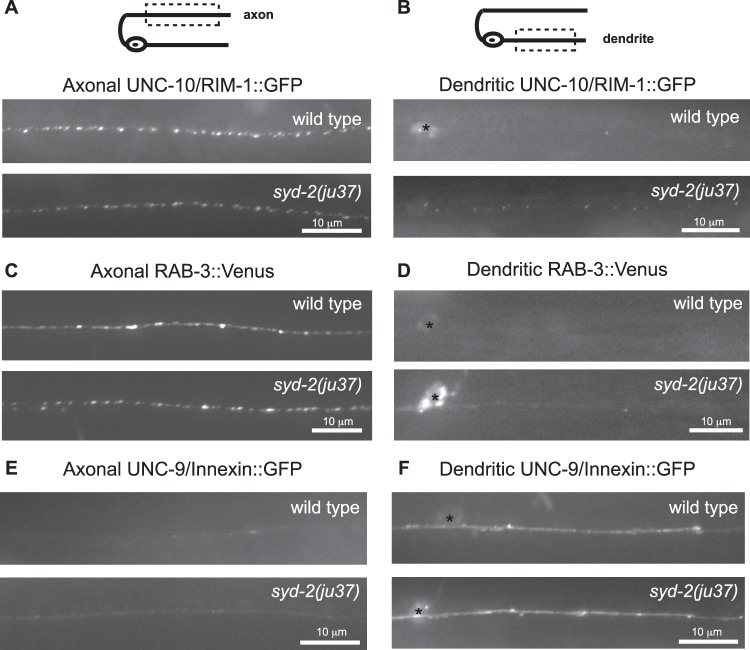
Analysis of axonal and dendritic markers in *syd-2* mutants. (A) Schematic diagram of a DB motor neuron (top panel). The boxed region denotes that the axon was imaged in panels (A, C, E). Representative images of active zone protein UNC-10/Rim-1::GFP in the axons of wild type and *syd-2(ju37)* mutant animals (bottom panels). (B) Schematic diagram of a DB motor neuron (top panel). The boxed region denotes that the dendrite was imaged in panels (B, D, F). Representative images of active zone protein UNC-10/Rim-1::GFP in the dendrites of wild type and *syd-2(ju37)* mutant animals (bottom panels). (C) Representative images of synaptic vesicle protein RAB-3::Venus in the axons of wild type and *syd-2(ju37)* mutant animals (D) Representative images of synaptic vesicle protein RAB-3::Venus in the dendrites of wild type and *syd-2(ju37)* mutant animals. (E) Representative images of UNC-9/Innexin::GFP in the axons of wild type and *syd-2(ju37)* mutant animals. (F) Representative images of UNC-9/Innexin::GFP in the dendrites of wild type and *syd-2(ju37)* mutant animals.

Changes in the polarized distribution of DCVs observed in *syd-2* mutants could be due to defects in synapse formation or neuronal polarity. Although SYD-2 is essential for active zone assembly and clustering of SVs at synapses in HSN neurons [36], [37], the absolute requirement for SYD-2 for presynaptic differentiation may vary depending on the neuronal cell type [35], [50]. In order to investigate the role of *syd-2* in synapse formation and neuronal polarity in DB neurons, we examined the distribution of two axonal markers, the active zone protein UNC-10/RIM-1::GFP and the synaptic vesicle-associated protein RAB-3::Venus [4], [55] in *syd-2* mutants. In wild type animals, UNC-10::GFP and RAB-3::Venus (under control of the *Punc-129* promoter) are localized to presynaptic sites in motor neuron axons but not dendrites (Figure 4A–D) [4], [7], [26]. We found that although *syd-2* is not required for the localization of UNC-10::GFP or RAB-3::Venus to presynaptic sites in DB axons (Fig. 4A, C), we observed some mis-localization of UNC-10::GFP to dendrites (Fig. 4B) and increased accumulation of RAB-3::Venus in cell bodies (Fig. 4D) of *syd-2* mutants. These findings, together with the relatively normal density of DCVs in *syd-2* mutant axons (Fig. 1), suggest that the number of presynaptic sites is not dramatically altered in *syd-2* mutant DB neurons. These results are consistent with another study which showed that the fluorescence intensity and density of several presynaptic markers either did not change or decreased about 10–20% in DA/DB motor neuron axons of *syd-2* mutants [50]. Taken together, these studies suggest that SYD-2 is not absolutely required for presynaptic differentiation in DB motor neurons and suggests that SYD-2 likely functions redundantly with other proteins to regulate active zone assembly in these neurons.

We also examined the distribution of a dendritic marker, the invertebrate GAP junction innexin protein UNC-9::GFP [26] in *syd-2* mutants. We found that the normal dendritic localization of the innexin UNC-9::GFP in wild type motor neurons was unaltered in *syd-2* mutants (Fig. 4E, F), suggesting that *syd-2* mutants do not have a general defect in dendritic trafficking or identity. Together with our axonal marker analysis, these data suggest that *syd-2* mutants do not have gross defects in synapse development or axon and dendrite development in DB motor neurons.

### syd-2 Regulates the Trafficking of DCVs in DB Axons and Dendrites

**Figure 5:**
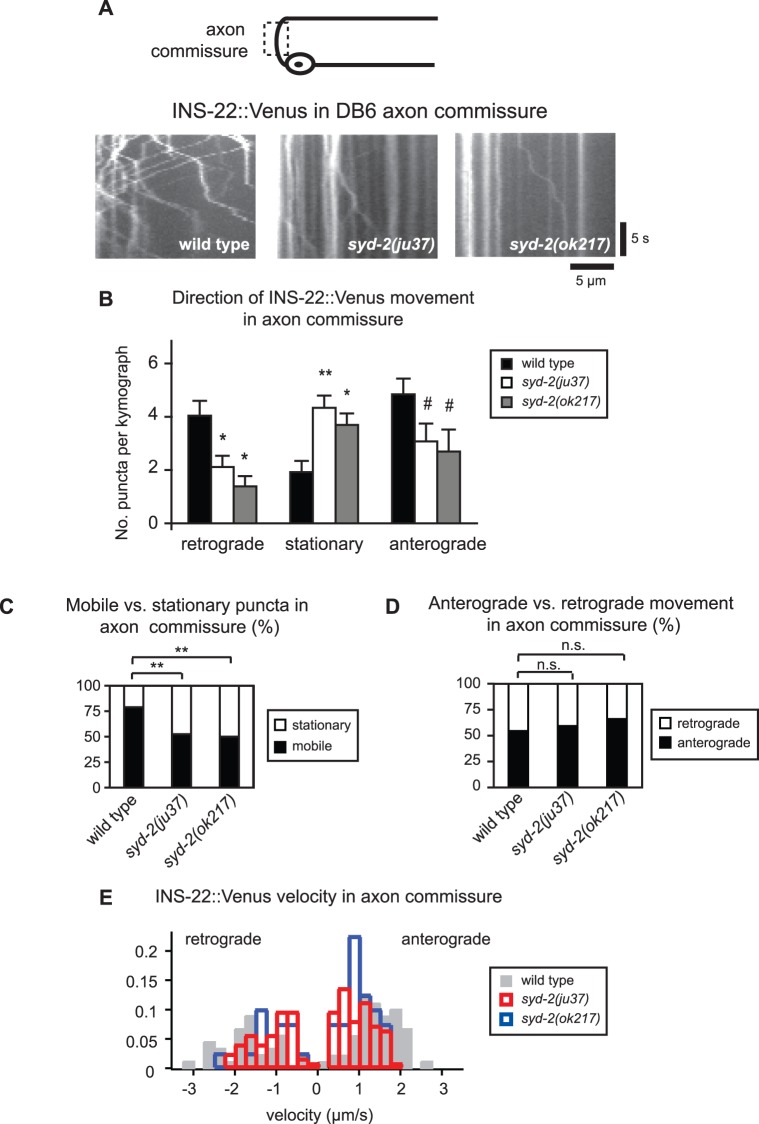
SYD-2 promotes the bi-directional movement of DCVs in the DB6 axon commissure. (A) Schematic diagram of a DB motor neuron (top panel). The boxed region denotes that the axon commissure was imaged for all the data presented in this figure. Representative kymographs from a 20 s movie of mobile INS-22::Venus puncta in the DB6 axon commissure of wild type, *syd-2(ju37)*, and *syd-2(ok217)* mutant animals (bottom panels). These kymographs are oriented with the cell body and ventral side to the left and the dorsal side to the right. (B) Quantification of the average number of INS-22::Venus puncta moving anterogradely, retrogradely, or remaining stationary in each kymograph from wild type (n = 25), *syd-2(ju37)*(n = 25), and *syd-2(ok217)*(n = 13) animals. (C) Quantification of the percentage of mobile and stationary INS-22::Venus puncta in the DB6 axon commissure of wild type (n = 227 puncta), *syd-2(ju37)* (n = 246 puncta), and *syd-2(ok217)* (n = 96 puncta) mutant animals. (D) Quantification of the percentage of anterograde and retrograde INS-22::Venus movement in the DB6 axon commissure of wild type (n = 179 puncta), *syd-2(ju37)*(n = 129 puncta), and *syd-2(ok217)*(n = 48 puncta) mutant animals. (E) Histogram of INS-22::Venus puncta velocities in the axon commissures of wild type, *syd-2(ju37)*, and *syd-2(ok217)* mutant animals. Positive values represent anterograde movements and negative values represent retrograde movements. Values that differ significantly (Student’s *t* test (B) and Chi Squared test (C-D)) from wild type are denoted on the graphs (# p≤0.05, *p≤0.01, **p≤0.001). Values that do not differ significantly (p>0.05) are denoted by n.s.

Since SYD-2 has been implicated in motor-dependent trafficking, we next tested whether SYD-2 is required for the anterograde movement of DCVs in axons. SYD-2/Liprin-α binds to motors such as UNC-104 and regulates their velocity and movement properties [42], [44], [45]. SYD-2/Liprin-α also regulates the motility of motor cargos such as the run length and directionality of SVs [43], [45]. We directly measured DCV movement using time-lapse microscopy and kymograph analysis of mobile DCVs in the DB6 axon commissures of wild type and *syd-2* mutants. The axon commissure is an asynaptic region of the axon connecting the cell body in the VNC to the presynaptic region of the axon in the DNC. Interestingly, we found that DCV trafficking in axon commissures was decreased in both the anterograde and retrograde directions in *syd-2(ju37)* and *syd-2(ok217)* mutant animals (Fig. 5A, B and Table 1). At the same time, we observed an increase in the number of stationary DCVs in the axon commissure in *syd-2* mutants compared to wild type controls (Fig. 5 and Table 1). The percentage of mobile versus stationary DCVs was significantly decreased in *syd-2* mutant axons (Fig. 5C and Table 1), whereas there was no change in the percentage of anterograde versus retrograde movement of DCVs (Fig. 5D and Table 1). Additionally, although we were unable to accurately measure run lengths in the axon commissure due to its curved trajectory, we found that DCV velocity was significantly reduced in both anterograde and retrograde directions in *syd-2(ju37)* and *syd-2(ok217)* mutant axons (Fig. 5E and Table 1). Because UNC-104 is required to traffic DCVs to presynaptic sites in axons [2], [4], these findings are consistent with reports that SYD-2 positively regulates the anterograde velocity of UNC-104 [45]. Interestingly, our data also show that SYD-2 regulates the velocity of DCVs moving in the retrograde direction, implicating SYD-2 as a positive regulator of bi-directional DCV trafficking.

**Table 1:** INS-22::Venus transport in DB axon commissures.

Direction	Avg. No. Anterograde^1^	Avg. No. Stationary^1^	Avg. No. Retrograde^1^	% Puncta Anterograde^2^	% Puncta Stationary^2^	% Puncta Retrograde^2^	Velocity (µm/s) Anterograde^3^	Velocity (µm/s) Retrograde^3^
Wild type	4.8±0.6	1.9±0.4	4.0±0.6	44.8	17.8	37.4	1.4±0.07	1.7±0.1
*syd-2(ju37)*	3.1±0.7 ^#^	4.3±0.5 **	2.1±0.4 *	32.3	45.5	22.2	0.9±0.05 **	1.1±0.07 **
*syd-2(ok217)*	2.7±0.8 ^#^	3.7±0.4 *	1.4±0.4 *	34.7	47.5	17.8	1.0±0.07 **	1.1±0.2

Data were generated from 20 second kymographs of wild type (n = 25), *syd-2(ju37)*(n = 25) and *syd-2(ok217)* (n = 13) mutant axons. Means ± SEM are reported.

1 Student’s t test used to compare significance of average number of puncta.

2 Chi square test used to compare significance of percentage of anterograde, stationary and retrograde puncta.

3 Kolmogorov-Smirnov test used to compare significance of puncta velocities (^#^p<0.05, *p<0.01, **p<0.001).

**Figure 6:**
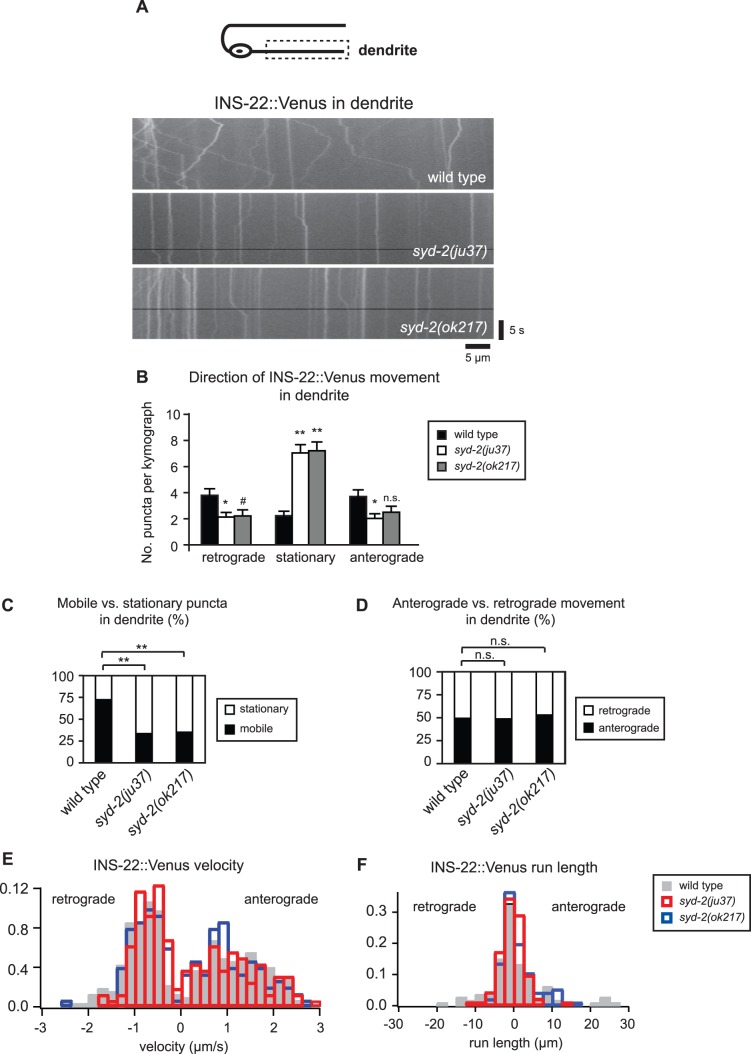
SYD-2 promotes the bi-directional movement of DCVs in DB dendrites. (A) Schematic diagram of a DB motor neuron (top panel). The boxed region denotes that the dendrite was imaged for all the data presented in this figure. Representative kymographs from a 20s movie of mobile INS-22::Venus puncta in the DB dendrites of wild type, *syd-2(ju37)*, and *syd-2(ok217)* mutant animals (bottom panels). (B) Quantification of the average number of INS-22::Venus puncta moving anterogradely, retrogradely, or remaining stationary in each kymograph from wild type (n = 44), *syd-2(ju37)*(n = 39), and *syd-2(ok217)*(n = 32) animals. (C) Quantification of the percentage of mobile and stationary INS-22::Venus puncta in the DB dendrites of wild type (n = 355 puncta), *syd-2(ju37)* (n = 414 puncta), and *syd-2(ok217)* (n = 356 puncta) mutant animals. (D) Quantification of the percentage of anterograde and retrograde INS-22::Venus movement in the DB dendrites of wild type (n = 257 puncta), *syd-2(ju37)* (n = 139 puncta), and *syd-2(ok217)* (n = 125 puncta) mutant animals. (E) Histogram of INS-22::Venus puncta velocities in the DB dendrites of wild type, *syd-2(ju37)*, and *syd-2(ok217)* mutant animals. Positive values represent anterograde movements and negative values represent retrograde movements. (F) Histogram of INS-22::Venus puncta run length in DB dendrites. Positive values represent anterograde movements and negative values represent retrograde movements. Values that differ significantly (Student’s *t* test (B) and Chi Squared test (C-D)) from wild type are denoted on the graphs (# p≤0.05, *p≤0.01, **p≤0.001). Values that do not differ significantly (p>0.05) are denoted by n.s.

Given that *syd-2* mutants have reduced DCV movement in DB axon commissures and an accumulation of DCVs in dendrites, we next tested whether SYD-2 also affects dendritic DCV trafficking. Similar to our findings in the axon commissure, we observed a large increase in the number of stationary puncta in the dendrites of *syd-2(ju37)* and *syd-2(ok217)* mutant animals (Fig. 6A, B and Table 2). We also observed a decrease in the number of DCVs moving in the anterograde and retrograde directions in *syd-2* mutant dendrites, although the decrease in anterograde DCVs observed in the *ok217* mutant allele did not reach statistical significance (Fig. 6B and Table 2). Similar to our findings in axons, we found that the percentage of mobile versus stationary DCVs was significantly reduced in *syd-2(ju37)* and *syd-2(ok217)* mutant dendrites (Fig. 6C), whereas the percentage of anterograde versus retrograde DCVs was not altered (Fig. 6D and Table 2). In addition, we found that *syd-2* mutants exhibit a small decrease in retrograde DCV velocity and reduced DCV run lengths in both directions in DB dendrites (Table 2). Taken together, our data support a role for SYD-2 as a general positive regulator of DCV movement in axon commissures and dendrites.

**Table 2:** INS-22::Venus transport in DB dendrites.

Direction	Avg. No. Anterograde^1^	Avg. No. Stationary^1^	Avg. No. Retrograde^1^	% Puncta Anterograde^2^	% Puncta Stationary^2^	% Puncta Retrograde^2^	Velocity (µm/s) Anterograde^3^	Velocity (µm/s) Retrograde^3^	Run length (µm) Anterograde^3^	Run length (µm) Retrograde^3^
Wild type	3.7±0.5	2.2±0.4	3.8±0.5	38.1	22.9	39.0	1.3±0.05	0.9±0.03	6.9±1.1	4.2±0.6
*syd-2 (ju37)*	2.0±0.4 *	7.0±0.6 **	2.1±0.4 *	18.1 **	62.9 **	19.0 **	1.2±0.08 n.s.	0.7±0.04 *	2.7±0.4 **	2.8±0.3
*syd-2 (ok217)*	2.5±0.5 n.s.	7.2±0.7 **	2.2±0.5 ^#^	20.5 **	61.1 **	18.4 **	1.1±0.08 n.s.	0.8±0.04 ^#^	3.2±0.5 *	3.1±0.5 ^#^

Data were generated from 20 second kymographs of wild type (n = 44), *syd-2(ju37)*(n = 39) and *syd-2(ok217)*(n = 24) mutant dendrites. Means ± SEM are reported.

1 Student’s t test used to compare significance of average number of puncta.

2 Chi square test used to compare significance of percentage of anterograde, stationary and retrograde puncta.

3 Kolmogorov-Smirnov test used to compare significance of puncta velocities and run lengths (^#^p<0.05, *p<0.01, **p<0.001, n.s. p>0.05).

### syd-2 also Regulates DCV Mobility in DA Motor Neurons

**Figure 7:**
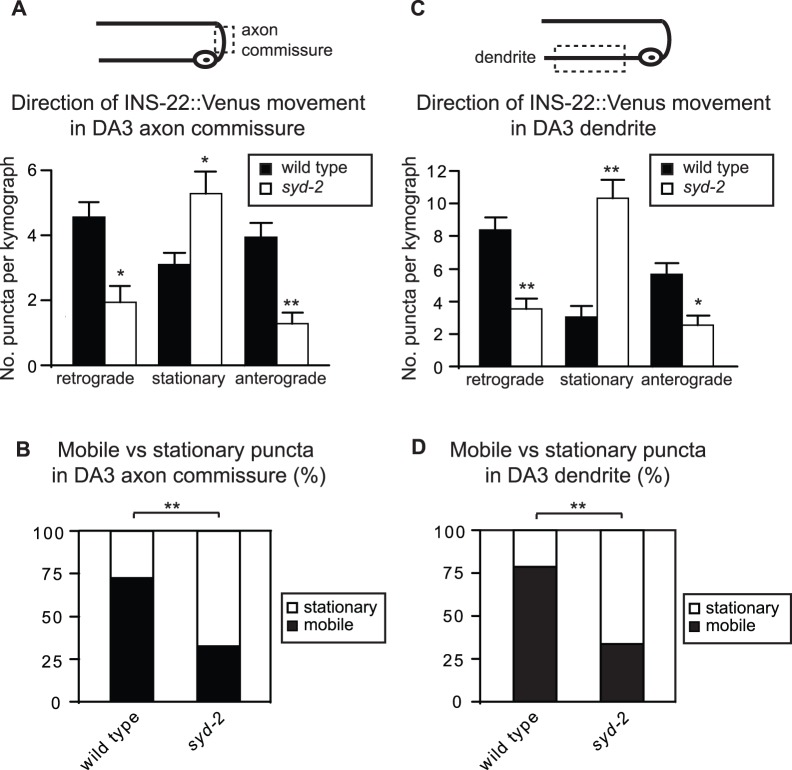
SYD-2 promotes the bi-directional movement of DCVs in DA3 axon commissures and dendrites. (A) Schematic diagram of a DA motor neuron (top panel). The boxed region denotes that the axon commissure was imaged for the data presented in panels (A–B). The graph (bottom panel) shows quantification of the average number of INS-22::Venus puncta moving anterogradely, retrogradely, or remaining stationary in kymographs generated from 20 s movies of mobile INS-22::Venus puncta in axon commissures of wild type (n = 29) and *syd-2(ju37)*(n = 18) mutant animals. (B) Quantification of the percentage of mobile and stationary INS-22::Venus puncta in the DA3 axon commissure of wild type (n = 325 puncta) and *syd-2(ju37)* (n = 141 puncta) mutant animals. (C) Schematic diagram of a DA motor neuron (top panel). The boxed region denotes that the dendrite was imaged for the data presented in panels (C-D). The graph (bottom panel) shows quantification of the average number of INS-22::Venus puncta moving anterogradely, retrogradely, or remaining stationary in kymographs generated from 20 s movies of mobile INS-22::Venus puncta in dendrites of wild type (n = 18) and *syd-2(ju37)*(n = 26) mutant animals. (D) Quantification of the percentage of mobile and stationary INS-22::Venus puncta in the DA3 dendrite of wild type (n = 257 puncta) and *syd-2(ju37)* (n = 405 puncta) mutant animals. Values that differ significantly (Student’s *t* test (A, C) and Chi Squared test (B, D)) from wild type are denoted on the graphs (# p≤0.05, *p≤0.01, **p≤0.001).

Interestingly, a previous study showed that the mechanisms involved in regulating the polarized trafficking of SVs can differ between DA and DB classes of cholinergic motor neurons [8]. To determine if SYD-2 regulates DCV movement in DA as well as DB class motor neurons, we performed time-lapse imaging of mobile DCVs in the DA3 axon commissure. Similar to our results from DB6 neurons, we found that *syd-2(ju37)* mutants exhibit increased amounts of stationary DCVs and decreased bi-directional DCV movement in DA3 axon commissures (Fig. 7A). Likewise, we found that loss of *syd-2* function reduced the percentage of mobile versus stationary DCVs (Fig. 7B), but not the ratio of anterograde to retrograde movement in DA3 axon commissures (ratio of anterograde/retrograde DCVs: wild type: 46%/54%; *syd-2(ju37)*: 40%/60%). Anterograde and retrograde DCV velocities were also reduced in DA3 commissures of *syd-2(ju37)* mutant animals (average anterograde velocity (µm/s): wild type: 1.07±0.04; *syd-2(ju37)*: 0.69±0.08; average retrograde velocity (µm/s): wild type: 1.45±0.05; *syd-2(ju37)*: 0.91±0.07).

Although we are not able to measure INS-22::Venus signal solely in DA dendrites due to overlap with DB dendrites, we can enrich for INS-22::Venus signal in DA neurons by imaging a section of the VNC where the vast majority of fluorescence signal comes from DA3 dendrites. However, we acknowledge that overlapping DB3 dendrites may make a minor contribution to the INS-22::Venus signal in this region due to occasional, weak expression of INS-22::Venus in DB3 neurons [7]. Time-lapse analysis of DCV movement in DA3 dendrites of *syd-2* mutants revealed a significant decrease in the number of mobile DCVs, an increase in the number of stationary DCVs, and a corresponding decrease in the percentage of mobile versus stationary DCVs (Fig. 7C, D). These results indicate that *syd-2* mutants have similar defects in DCV mobility in both DA and DB motor neurons suggesting that SYD-2 promotes DCV trafficking in both axon commissures and dendrites in both motor neuron classes.

### Dynein is Required for Increased Dendritic DCVs in syd-2 Mutants

**Figure 8:**
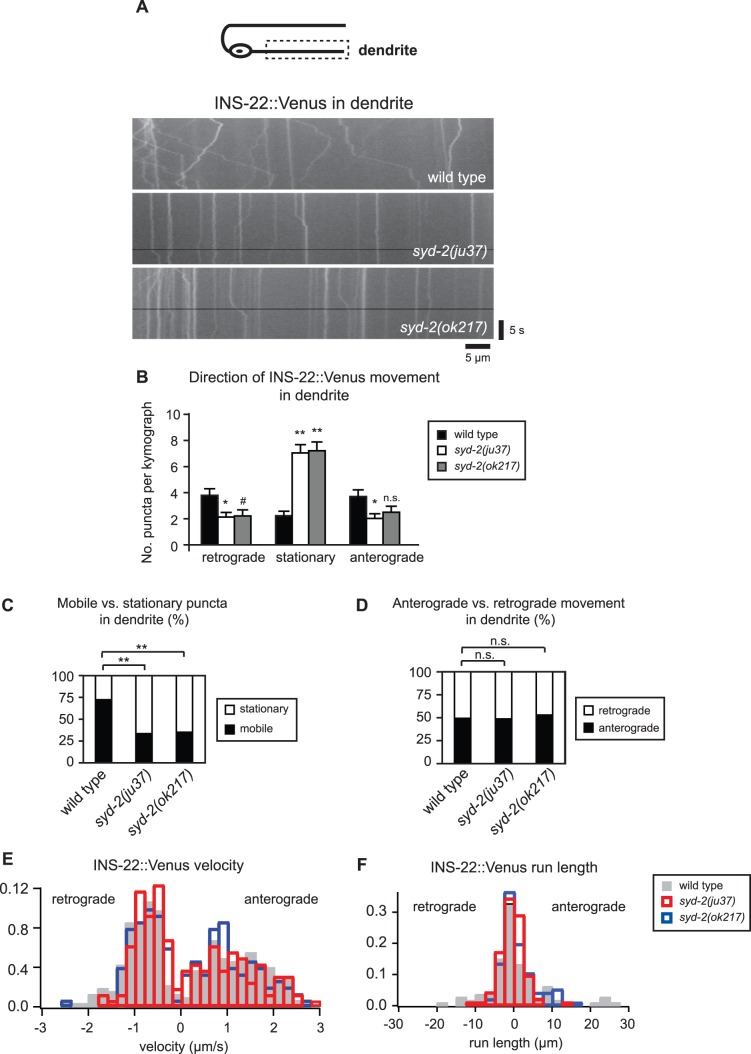
Dynein is required for the increase in dendritic DCVs in *syd-2* mutants. (A) Schematic diagram of a DB motor neuron (top panel). The boxed region denotes that the dendrite was imaged. Representative images of INS-22::Venus in the dendrites of wild type, *syd-2(ju37), dhc-1(js319),* and *syd-2;dhc-1* mutant animals. (B) Quantification of INS-22::Venus puncta density in the dendrites of wild type (n = 44), *syd-2(ju37)* (n = 30), *dhc-1*(js319) (n = 12), and *syd-2;dhc-1* (n = 16) mutant animals. Values that differ significantly (Tukey-Kramer) from wild type (marked by asterisks above each bar) or from other genotypes (comparisons marked by brackets) are denoted on the graphs (#p≤0.05, *p≤0.01, **p≤0.001). Values that did not differ significantly (p>0.05) are denoted by n.s.

Because *syd-2* mutants have decreased bi-directional movement of DCVs in the dendrite, yet still manage to accumulate more stationary DCVs, we investigated whether active transport of DCVs on microtubules was required for the increase in dendritic DCVs observed in *syd-2* mutants. We previously used EB1 plus-end binding protein dynamics to show that approximately 90% of the microtubules in DB dendrites are oriented minus-end out relative to the cell body and the microtubule minus-end directed motor dynein is required for anterograde trafficking of DCVs into dendrites [7]. In order to test whether dynein-mediated transport is also required for the increased dendritic localization of DCVs in *syd-2* mutants, we analyzed the distribution of INS-22::Venus in *syd-2;dhc-1(js319)* double mutant dendrites. We found that the *dhc-1(js319)* loss of function mutation blocked the increase in INS-22::Venus puncta density observed in *syd-2(ju37)* mutants (Fig. 8). This result suggests that dynein is required for the increased accumulation of DCVs in *syd-2* mutant dendrites.

## Discussion

### SYD-2 Regulates DCV Movement

Neuropeptide-containing DCVs can be localized in a polarized manner to axons but the molecules involved are not well defined. Here, we show that the scaffolding protein SYD-2/Liprin-α is required for the normal polarized localization of DCVs to axons in cholinergic DA and DB motor neurons. The polarized localization of two DCV markers, neuropeptides INS-22::Venus and NLP-21::Venus, to axons is disrupted in *syd-2* loss of function mutants (Fig. 1). Time-lapse microscopy revealed that *syd-2* mutants have several defects in DCV movement in DB motor neurons. We found that the number of DCVs moving in both the anterograde and retrograde directions is decreased in axon commissures and dendrites in two independent *syd-2* loss of function mutants (Figs. 5 and 6). This decrease in DCV movement was combined with a 2–3 fold increase in the number of stationary DCVs resulting in a decrease in the percentage of mobile versus stationary DCVs in *syd-2* mutants (Figs. 5 and 6). We also found that DCV velocities were reduced in *syd-2* mutant axon commissures and dendrites and that DCV run lengths were decreased in *syd-2* mutant dendrites (Tables 1 and 2). Finally, we found that the effects of *syd-2* on DCV trafficking were not specific to DB motor neurons, because we observed similar reductions in DCV movement in DA motor neurons (Fig. 7). These results indicate that SYD-2 promotes bi-directional DCV mobility in both axon commissures and dendrites of DA and DB motor neurons.

### Comparison of SYD-2 Effects on DCV and SV Trafficking

Several studies have shown that SYD-2/Liprin-α can bind motors and regulate the movement of motors or their SV cargo [42]–[45]. Studies in *Drosophila* showed that Liprin-α interacts with the heavy chain of kinesin-1 *in vitro* and promotes the anterograde movement of SVs in axons [43]. Specifically, *liprin-α* mutants exhibit decreased anterograde flux of SVs in axons due to decreased anterograde processivity and increased initiation of retrograde movement [43]. In *C. elegans*, SYD-2 binds and clusters the SV motor UNC-104/KIF1A and promotes its anterograde movement and velocity, resulting in a decrease in net retrograde movement of SVs [45]. Our data show that SYD-2 promotes anterograde DCV trafficking and velocity in axon commissures of DB neurons (Fig. 5 and Table 1). Because UNC-104 traffics DCVs to presynaptic sites in DA/DB motor neuron axons [2], [4], [5], [7], our data are consistent with the idea that SYD-2 promotes anterograde trafficking of DCVs on UNC-104 in axon commissures. Our data also show that SYD-2 promotes retrograde DCV trafficking in axon commissures (Fig. 5) revealing a novel role for SYD-2 in regulation of retrograde transport.

There are multiple ways in which SYD-2 could regulate the retrograde trafficking of DCVs. We previously showed that microtubule orientation is predominantly plus-end out relative to the cell body in axons and largely minus-end out in dendrites of DB motor neurons in *C. elegans* [7]. In the same study, we showed that the minus-end-directed microtubule motor dynein is required for anterograde trafficking of DCVs in these dendrites and likely also for retrograde trafficking in axons [7], consistent with a prior study showing that dynein transports SVs retrogradely in DA motor neuron axons [8]. These studies suggest that dynein transports DCVs retrogradely in axons and anterogradely in dendrites. Because we find that *syd-2* mutants have reduced bi-directional trafficking of DCVs, our data could implicate SYD-2 in regulating dynein-mediated trafficking of DCVs. Consistent with this idea, we found that mutations in dynein heavy chain, *dhc-1*, block the increased accumulation of DCVs in *syd-2* mutant dendrites (Fig. 8). However, because a previous study failed to observe an interaction between SYD-2 and dynein [43], it remains possible that SYD-2 regulation of dynein is not direct.

SYD-2 might regulate retrograde trafficking of DCVs indirectly via its effects on anterograde trafficking. Multiple recent studies suggest that anterograde and retrograde movement of DCVs are tightly coupled. Mutation of the anterograde motor *unc-104/kinesin-3* disrupts both the anterograde and retrograde flux of DCVs in *Drosophila* [1]. Conversely, inhibition of the dynactin complex, which is required for dynein function, disrupts anterograde as well as retrograde trafficking [56]. Finally, by tracking individual DCVs throughout multiple sections of the axon, Wong et al (2012) demonstrated that DCVs undergo extensive long-range recirculation in axons, which may explain why disruption of DCV trafficking in one direction affects movement in the other direction [57].

Here we show that SYD-2 regulates two aspects of DCV trafficking: DCV mobility and the polarized localization of DCVs to axons versus dendrites. How loss of DCV mobility in *syd-2* mutants could result in a loss of DCVs from presynaptic sites in the axon and a simultaneous increase in dendrites is not yet clear. One possible model is that in *syd-2* mutants, the decrease in DCV mobility results in fewer DCVs reaching presynaptic sites due to an increase in stationary DCVs in the axon commissure and cell body. The accumulation of DCVs in the cell body might then lead to increased numbers of DCVs in the dendrite over time. This model is consistent with our previous data showing that in *unc-104* mutants, DCVs are not trafficked to the axon but instead accumulate in the cell body and dendrite [7]. However, we cannot exclude the possibility that SYD-2 regulates the polarized localization of DCVs and bi-directional DCV trafficking through independent mechanisms. It remains possible that the change in the polarized distribution of INS-22::Venus in *syd-2* mutants is due to compartment-specific changes in DCV release (i.e., more release from axons and less release from cell bodies and dendrites), however it has not yet been shown whether or not DCVs can be released from cell bodies and dendrites of DA/DB motor neurons. Further studies of DCV localization during development will be needed to determine if accumulation of DCVs in *syd-2* mutant cell bodies precedes dendritic DCV accumulation and whether SYD-2′s effects on DCV polarity are directly due to changes in DCV mobility.

Finally, SYD-2 may not directly regulate motor movement but might regulate the ability of DCVs to associate with motors. SYD-2 has been proposed to function as an adaptor to link cargo to motors. For example, Liprin-α was shown to be degraded in a CaMKII-dependent manner in hippocampal neurons and this regulated degradation was proposed as a mechanism to promote cargo unloading at active synapses [47]. Intriguingly, CaMKII has been shown to promote the synaptic capture of trafficking DCVs in *Drosophila* neurons [58], [59]. Our data are also consistent with a role for SYD-2 in promoting DCV attachment to motors. In this scenario, DCVs are unloaded prematurely in *syd-2* mutant axons and dendrites, resulting in decreased run lengths and increased numbers of stationary DCVs. While this is an attractive model, the decreased DCV velocities observed in *syd-2* mutants suggest that SYD-2 may also regulate the movement properties of DCV-motor complexes. It will be interesting in the future to test whether synaptic activity and CaMKII regulate SYD-2 degradation and DCV unloading from motors such as UNC-104 or dynein.

### Role of SYD-2 in Synapse Development Versus Intracellular Trafficking

SYD-2/Liprin-α appears to have multiple functions in neurons. SYD-2/Liprin-α is essential for active zone assembly in *C. elegans* HSN neurons and regulates active zone morphology in *C. elegans* GABAergic neurons and at the Drosophila NMJ [31]–[33], [35]–[37]. SYD-2/Liprin-α also interacts with microtubule motors and regulates intracellular trafficking of pre- and postsynaptic cargo [43]–[45], [48], [49].

Although *syd-2* plays an essential role in synapse formation in HSN neurons [36], [37], studies in other neuronal cell types suggest that SYD-2 is not absolutely required for active zone assembly and clustering of SVs [35], [50], suggesting that in some cases SYD-2 functions with other molecules to regulate presynaptic development. While *syd-2* does not appear to be required for clustering of active zone and SV markers in DA/DB motor neurons, we found a slight mislocalization of UNC-10::GFP to dendrites (Fig. 4) and Ch’ng et al. (2008) found a 20% decrease in UNC-10::GFP fluorescence in axons suggesting that SYD-2 contributes to active zone assembly in these neurons [50]. However, loss of synaptic UNC-10 itself is unlikely to be a direct cause of DCV polarity defects because *unc-10* loss of function mutants do not have defects in DCV localization [7]. Thus, although the effects of SYD-2 on synapse formation may contribute to DCV polarity defects in DB motor neurons, SYD-2 does not appear to play a major role in synapse formation in DB neurons. Instead, our data identify SYD-2 as a novel regulator of bi-directional DCV mobility and the polarized distribution of DCVs. It will be interesting in the future to determine if SYD-2 regulates other motors and cargos and whether it plays a broader role in regulating intracellular transport.
