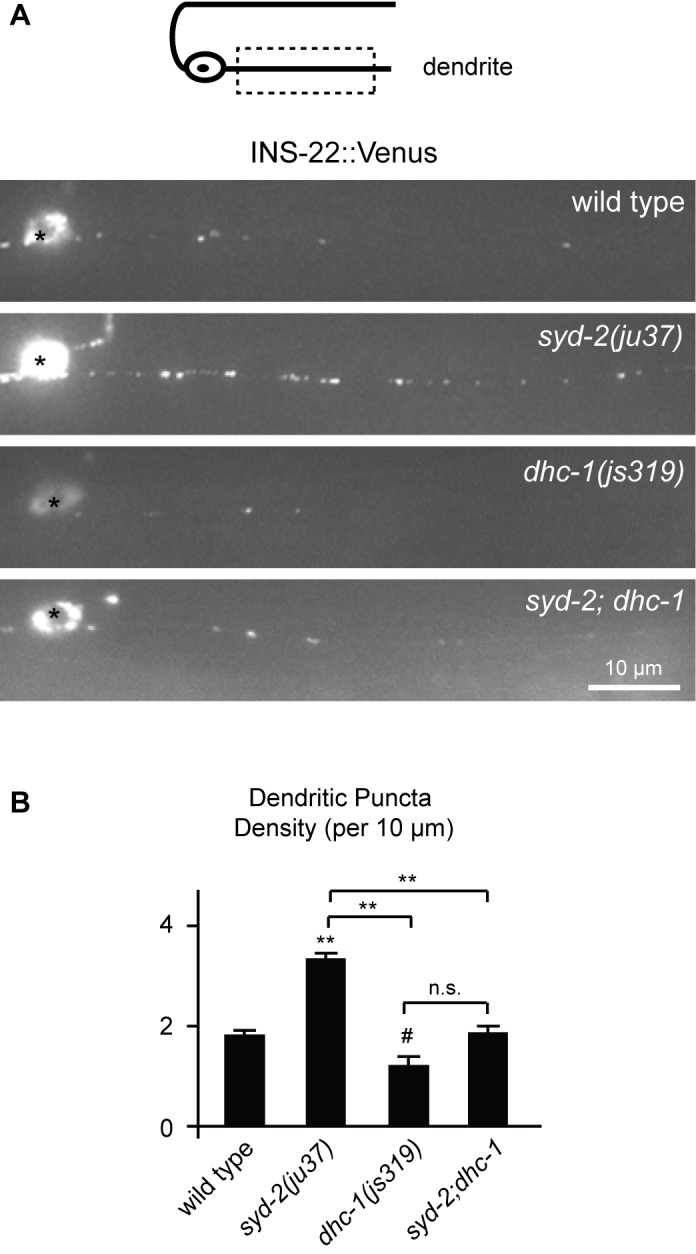# Correction: The Scaffolding Protein SYD-2/Liprin-α Regulates the Mobility and Polarized Distribution of Dense-Core Vesicles in *C. elegans* Motor Neurons

**DOI:** 10.1371/annotation/dbc0d5dd-ab9b-4ae8-b5cf-507158f0761f

**Published:** 2013-02-27

**Authors:** Patricia R. Goodwin, Peter Juo

There was an error with Figure 8 - it was a duplicate of Figure 6.

The correct version of Figure 8 can be viewed here: 

**Figure:**